# Comparative study of salivary pH and volume in adults with chronic laryngopharyngitis by gastroesophageal reflux disease before and after treatment

**DOI:** 10.1016/S1808-8694(15)30035-5

**Published:** 2015-10-19

**Authors:** Claudia Alessandra Eckley, Henrique Olival Costa

**Affiliations:** a PhD, Professor from the School of Medical Sciences of the Santa Casa de SP – Assistant Professor – Department of Otolaryngology - ISCMSP; b PhD, Professor from the Medical School of the University of São Paulo – Assistant Professor – Otolaryngology - ISCMSP Otolaryngology Department – Santa Casa de São Paulo

**Keywords:** GERD, LPR, saliva, pH, volume

## Abstract

**Introduction:**

Gastroesophageal Reflux Disease (GERD) is the most prevalent digestive disease of the modern society and has been associated with abnormalities in the larynx and pharynx (LPR). Nonetheless, little is known about the mechanisms involved in this atypical form of the disease. Contradictory clinical data suggest a defense deficit at this segment. Saliva with its organic and inorganic components is responsible for the homeostasis of the oral mucosa and the digestive tract. Salivary pH and volume abnormalities have been linked to laryngopharyngeal symptoms of GERD and LPR. In a recent study we demonstrated significant salivary pH reduction in patients with LPR. Another study found correlation between reduced salivary pH and volume directly related to esophageal pH-metry results.

**Aim:**

To evaluate salivary pH and volume before and after clinical treatment of LPR.

**Material and Method:**

Twenty-three adults with LPR had total fasting saliva tested before and after a 12-week course of oral proton pump inhibitor.

**Results:**

A statistically significant difference was found in salivary pH before and after treatment with increase of pH values after control of the disease (p<0.001). Salivary volumes of treated patients were also significantly higher than in pre-treated patients (p=0.009).

**Discussion:**

These findings suggest that salivary pH and volume are influenced by the presence of gastroesophageal contents and that salivary pH monitoring can potentially become a cost-effective method for diagnosing and controlling LPR.

## INTRODUCTION

Gastroesophageal reflux (GER) is characterized by the spontaneous movement of the stomach gastric content to the esophagus. This reflux occurs daily in all human beings, being asymptomatic and without damage to the esophageal mucosa, thus considered physiologic. Notwithstanding, GER may cause great morbidity when it becomes symptomatic and causes lesions. Under these circumstances, it is known as Gastroesophageal reflux disease (GERD), being caused by a combination of reflux contact irritating factors (specially chloric acid and pepsin) with the mucosa and a deficiency in mucosal protection mechanisms. The Gastroesophageal Reflux Disease is considered the most prevalent gastrointestinal disease today. Reflux-related symptoms are reported weekly by 3 to 6% of the general population^1^, ^2^, ^3^, ^4^, ^5^, ^6^, ^7^, ^8^, ^9^. Multicentric studies with large numbers of people have shown how this disease may manifest in a silent or a multisymtomatic way from the digestive stand point^7^, ^8^, ^9^. Notwithstanding, its symptoms cause important drops in the individual's life quality7. It is believed that this great prevalence is due to a combination of a number of factors, from bad feeding habits and obesity^10^, ^11^ all the way down to genetic factors^9^.

In the last 12 years, many investigators and clinicians have proposed an association between Gastroesophageal Reflux Disease (GERD) and chronic laryngitis, the later representing the clinical form of the disease called Laryngopharyngeal Reflux (LPR)^1^, ^2^, ^3^, ^4^, ^8^, ^12^, ^13^, ^14^, ^15^, ^16^, ^17^, ^18^, ^19^, ^20^, ^21^, ^22^, ^23^, ^24^, ^25^, ^26^, ^27^, ^28^, ^29^, ^30^. The larynx findings in cases of reflux laryngitis vary according to the case severity, it goes from hyperemia and mild edema of the larynx posterior third to severe cases of contact ulcers in the vocal process, exuberant scar tissue, larynx lining alterations, subglotic stenosis, even neoplastic degeneration of the epithelium^14^, ^31^, ^32^.

According to some authors, estimates as to acid reflux causing posterior laryngitis vary greatly, coming up to 80% of the cases^14^, ^33^. This causal relationship has increased thanks to the technological development of devices capable of measuring the proximal and distal stomach acidity and that of the pharynx^13^, ^21^, ^34^, ^35^, ^36^, ^37^, ^38^, ^39^, ^40^, we also have optic fibers which are broadly used in clinical practice, and they do facilitate viewing the larynx^19^, ^20^. We still do not know, exactly, how many reflux episodes are necessary to produce inflammatory changes and larynx lesions. Notwithstanding, studies such as the ones from Delahunty & Cherry in 1968^41^ and Koufman in 1991^14^ showed that the attack of chloric acid and pepsin on the laryngeal mucosa of dogs caused contact granulomas in the first study, and subglotic stenosis in the second study, after some weeks. The role of non-acid substances, such as bile and pancreatic secretions, in causing larynx lesions is still controversial because we still lack broadly available techniques to clinically measure it^33^, ^42^. It is interesting to notice how a large number of laryngopharyngeal reflux patients, even those with the most dramatic larynx findings, do not have esophagitis or other signs of GERD in their digestive tract^8^, ^25^, ^29^. Of course, the gastric and esophageal mucosas protection mechanisms play a decisive role in the capacity these organs have to withstand daily mechanical and chemical aggressions, and many of these mechanisms are saliva mediated^31^, ^32^, ^45^, ^46^, ^47^, ^48^. Saliva has many organic and inorganic substances that contribute to the protection against physical and chemical aggression and also to maintain mucosal integrity, not only of the oral cavity, but also of the digestive tract^43^, ^45^, ^46^, ^48^, ^49^, ^50^, ^51^, ^52^.

We then set sail, based on the following assumptions:

• The esophageal-salivary reflex increases salivary production (both qualitatively and quantitatively), with a consequent increase in its presence within the esophagus, when there is any mechanical stimulation in this organ^43^, as is the case with laryngopharyngeal reflux. Thus, there is a greater availability of organic and inorganic salivary substances in the esophagus, besides the salivary volume itself that, together contribute to dilute and neutralize the refluxed contents^43^, ^45^, ^46^, ^48^.
• Recent studies in patients with LPR have shown a reduction in the salivary pH of these patients when compared to normal individuals without the disease^25^, ^31^, ^53^, ^54^. It was also noticed that there is a positive correlation between the presence of laryngopharyngeal symptoms and a reduction both in volume and in saliva pH in LPR patients^53^, it is possible to establish a correlation between the presence of esophageal reflux measured through 24h esophageal pHmetry and this reduction in salivary volume and pH^54^.
• Recent population studies show that over 30% of the general population present occasional laryngopharyngeal symptoms related to gastroesophageal reflux and up to 40% of these individuals have normal endoscopic exam of their digestive mucosa^8^. These findings point towards the GERD silent behavior, but also lead us to infer that there may possibly be more factors involved in the GERD physiopathology than only the gastroduodenal reflux, being acid or alkaline.

LPR is known as a risk factor for the development of a number of laryngopharyngeal mucosa alterations, and the most severe is the neoplastic degeneration^14^.

Despite the progress in understanding GERD and LPR, there are still many unanswered questions, specially in regards to the latter. We still do not know why patients with the same degree of proximal gastric reflux (to the larynx and pharynx) present manifestations and clinical findings of such different magnitude. Could it be some local larynx and pharynx mucosa protection factor that could be deficient or simply did not exist, making certain individuals so sensitive to this reflux? Eckley's study (2002)^25^ suggests that there is, in fact, a deficit of epidermal growth factor (EGF) in the salivary secretion, such substance is produced by the salivary glands, besides a significant alteration in the salivary pH of individuals with chronic laryngitis due to LPR, when compared to their normal counterparts. Notwithstanding, we do not know if these salivary alterations, both quantitative and qualitative, are innate or if they develop along one's life span. We also do not know if controlling GERD and LPR allow for a balancing on these salivary alterations.

Our goal with this study is to assess both the salivary volume and pH in LPR individuals before and after controlling the disease in order to establish whether the previously seen deficit is primary or secondary.

## MATERIALS AND METHODS

We studied 19 individuals with clinical videolaryngoscopy LPR diagnosis corroborated by a positive two channel 24 hour esophageal pHmetry. The participants in the study answered a detailed questionnaire about their general health and GERD-related symptoms, its digestive and otolaryngological consequences. All the study participants were examined by means of a flexible nasofibroscope according to previously established protocol 25, done right at the beginning of the study (pre-treatment exam) and after clinical treatment (post-treatment exam).

The 19 participants in the study had two total spontaneous saliva sample collected in the morning after a 12 hour fasting period, one in the pre-treatment phase and another in post treatment. The patients were instructed not to use toothpaste on the harvesting day, which was carried out according the following technique:

### Harvesting of Total Spontaneous Saliva

After rinsing the mouth with plain water to flush away epithelial scaling and bacterial remains, the patient remained seated, without swallowing saliva for a period of 15 minutes. This saliva would then run to the corner of the mouth (the subject should also avoid bringing nasal material into his/her mouth) into a glass funnel, linked to a test tube.

### Saliva processing

Total saliva was centrifuged for 10 minutes in room temperature at a speed of 5,000-7,000 revolutions per minute (rpm) in order to sediment bacteria, epithelial cells, nucleus remains and other debris. The supernatant was collected with a pipette and transferred to an ml graded Falcon tube in order to measure volume and pH. The samples were alpha-numerically labeled.

### Measuring Saliva Volume

In order to determine saliva volume, we used supernatant saliva, free from debris and foam. Measurement was carried out through the Falcon test tube grading itself, and volume was recorded in ml.

### Measuring Saliva pH

Salivary pH (supernatant saliva previously processed by the aforementioned technique) was digitally measured through a digital pH sensor (Denver Instrument Company, Model: Basic pH-meter; Arvada, CO, USA). We initially calibrated the device using buffered solutions with pH 4.0 and pH 7.0. after that the sensor probe was dipped in saliva filled Falcon tube, where it remained for 30 seconds, thus yielding automatic pH reading.

### LPR Treatment

Treatment was initially clinical and standardized for all patients, being:

Omeprazole 20mg per os before breakfast and 20mg per os after dinner for 12 (twelve) weeks.

After this time, the patients were reassessed from the stand point of clinical symptoms and laryngoscopic signs of LPR. Those who presented clinical control of symptoms and of the laryngitis signs had their saliva harvested^7^ days after medication interruption. We compared both the saliva volume and pH before and after treatment, as well as the correlation of symptoms relief and larynx inflammatory findings. Results were put in a table and statistically analyzed.

### Inclusion Criteria

We only included in the study those patients that had videolaryngoscopy diagnosis of LPR corroborated by two channel 24h esophageal pHmetry, who consented their participation after being fully informed about our goals, procedures used and risks involved.

### Exclusion Criteria

Exclusion factors were: smoking, alcohol drinking and exposure to abrasive chemical inhalants, because all these factors cause respiratory mucosa inflammation, thus mimicking the changes found in the GERD. Besides, we also excluded the patients who had used gastric secretion blocking agents, pro-kinetic, anti-acids or hormonal and non-hormonal anti-inflammatory agents in the 7 days preceding salivary harvesting, because of the influence these drugs have on the digestive tract mucosa and in gastric secretion. We also excluded patients with pre-neoplastic or neoplastic lesions of the pharynx and larynx (present or previously treated). Individuals with omeprazole intolerance were excluded, as well as those patients who did not present disease control after the established treatment period for this study.

## RESULTS

A total of 19 patients were eligible to participate in the study, 14 women and 5 men, with average age of 43.7 years, ranging from 19 to 56 years. All had videolaryngoscopy suggestive signs of moderate degree LPR^31^.

**Figure 1:**
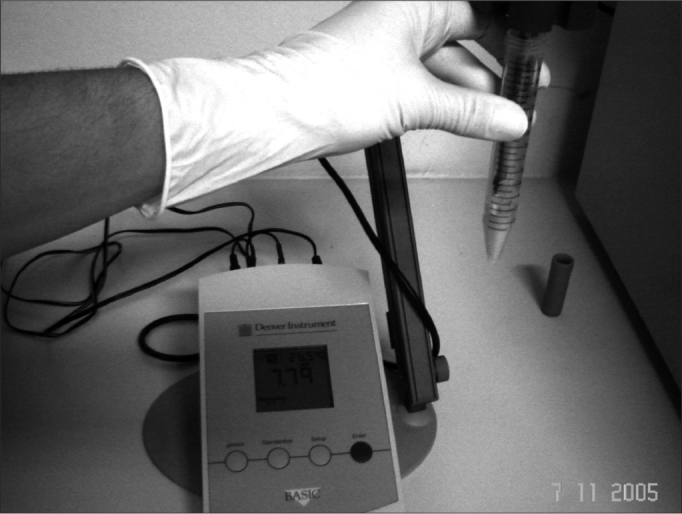
Falcon graded test tube with saliva supernatant. Salivary pH digital measuring method demonstration.

**Figure 2:**
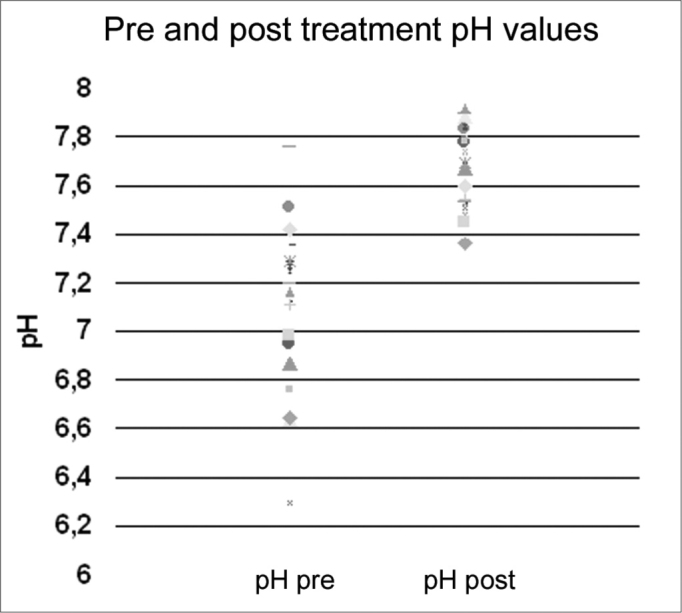
Salivary pH values pre and post treatment and LPR clinical control.

**Figure 3:**
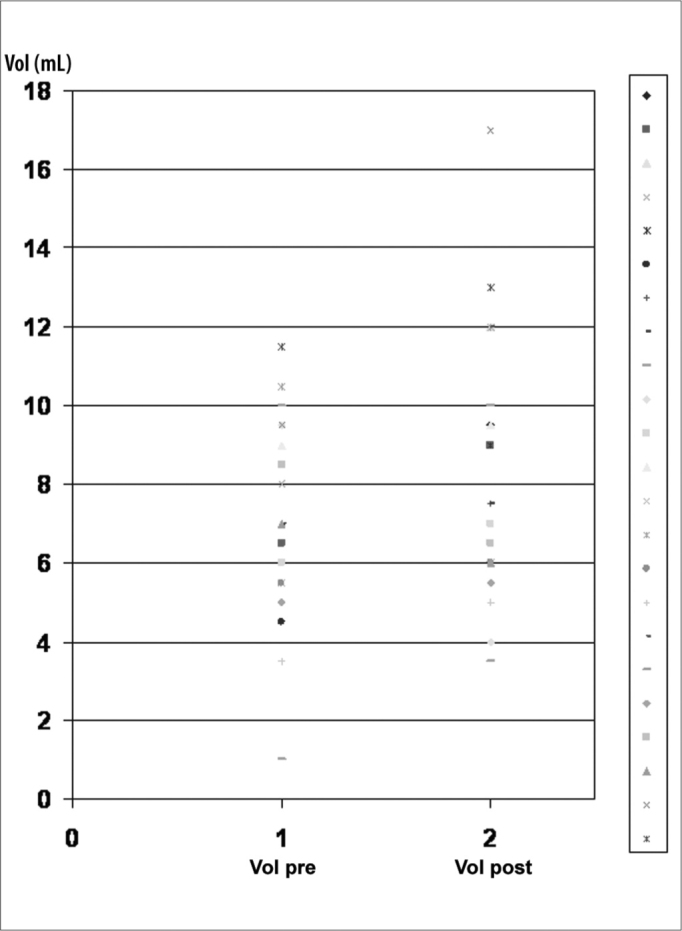
Pre and post treatment Salivary volume values and LPR clinical control.

The average pre-treatment salivary pH of the group was 7.15 and post-treatment was 7.58, statistically significant difference (p<0.05). Average pre-treatment salivary volume was of 7.07ml comparing to a value of 8.02ml post-treatment – there was no statistically significant difference between the samples, although we did see a trend towards an increase in salivary volume after treatment (Table 1).

**Table 1:** Clinical, laryngoscopic and salivary data from the participants in the study.

Patient #	Gender	Age	LPR Class	PH Pre	Vol. pre (mL)	PH post	Vol. post (mL)
1	F	56	MOD	7,29	9,5	7,68	9,5
2	F	60	MOD	6,98	6,5	7,79	9
3	M	46	MOD	6,87	9	7,67	12
4	F	47	MOD	6,95	8	7,48	12
5	M	24	MOD	7,25	8,5	7,88	9
6	F	19	MOD	6,95	4,5	7,78	7
7	F	41	MOD	7,29	6	7,69	7,5
8	M	43	MOD	7,12	7	7,52	7,5
9	F	36	MOD	7,76	1	7,89	3,5
10	F	50	MOD	7,42	9,5	7,6	4
11	F	21	MOD	6,98	6	7,45	7
12	F	44	MOD	6,64	9	7,88	9,5
13	F	56	MOD	7,25	5,5	7,74	6
14	F	47	MOD	7,29	10,5	7,69	12
15	F	50	MOD	7,51	5,5	7,83	6
16	F	48	MOD	7,11	3,5	7,54	5
17	F	48	MOD	7,35	10	7,63	12
18	M	46	MOD	7,2	10	7,53	10
19	M	48	MOD	6,65	5	7,36	5,5
20	F	47	MOD	6,76	8,5	7,78	6,5
21	F	72	MOD	7,16	7	7,92	6
22	F	56	MOD	6,3	9,5	7,51	17
23	F	41	MOD	7,27	11,5	7,84	13

There was no statistical correlation between salivary pH or volume related to gender, age or intensity of complaints, either laryngopharyngeal or digestive.

When we compared salivary pH values of the individuals with LPR with those from the pre-established group of normal individuals^25^, we noticed that even having a significant increase in values after disease treatment and control, salivary pH remained below the average of that from the normal individuals (p=0.04).

All patients tolerated well the proposed treatment, without side effects.

## DISCUSSION AND CONCLUSIONS

The few studies in the world medical literature mention qualitative and quantitative changes in the saliva of patients with GERD DRGE^45^, ^46^, ^48^, ^55^, ^56^ and LPR^31^, ^32^, ^54^, but did not ascertain whether these alterations were congenital or acquired. These and other studies indicate that the laryngeal mucosa is very sensitive to gastroduodenal reflux, and suggest that LPR, even when intermittent, may cause severe larynx inflammatory lesions^1^, ^30^. Notwithstanding, so far we were unable to establish the factors that influence the intensity of symptoms with the laryngoscopic findings, and were also unable to explain the lack of compatibility of such findings. Salivary studies in these individuals have shown new horizons in understanding LPR physiopathology, suggesting that there are both qualitative and quantitative saliva deficits^25^, ^31^, ^32^, ^53^, ^54^.

The results from the current study suggest that both LPR treatment and clinical control allow for a reduction in salivary acidity, or better, an alkalinization closer to physiological levels, because adult saliva pH is of about 8, and in LPR individuals it is closer to 7.0-7.5. Despite the small size sample in this study (which is still ongoing), we believe that there is global salivary deficit, possibly primary in LPR individuals, as we have proven in previous studies of volume, pH and organic substances such as epidermal growth.

The salivary pH study offers a less invasive way to assess local acidity, it is fast and of low cost.

## References

[1] Cianci R, Fedeli G, Cammarota G, Galli J, Agostino S, Di Girolamo S, Maurig M, Gasbarrini G. (2000). Is the alkaline reflux a risk factor for laryngeal lesions?. Am J Gastroenterol.

[2] Hanson DG, Jiang JJ (2000). Diagnosis and management of chronic laryngitis associated with reflux. Am J Med.

[3] García-Compéan D, Gonzalez GG, Mar DA, Trevino RM, Bosques F, Maldonado H. (2000). Prevalence of gastroesophageal reflux disease in patients with extraesophageal symptoms referred from otolaryngology, allergy, and cardiology practices: a prospective study. Dig Dis.

[4] Reulbach TR, Belafsky PC, Blalock D, Koufman JA, Postma GN (2001). Occult Laryngeal Pathology in a Community-Based Cohort. Otolaryngol. Head & Neck Surg.

[5] Moraes-Filho JPP, Cecconello I, Gama-Rodrigues J, Castro LP, Henry M,A, Meneghelli UG, Quigley E. (2002). Brazilian consensus on gastroesophageal reflux disease: proposals for assessment, classification, and management. Am J Gastroenterol.

[6] Anelli W. Aspectos perceptivo-auditivos e acústicos da doença do refluxo gastroesofágico. São Paulo, 2002. (Tese – Mestrado – Escola Paulista de Medicina)

[7] Kulig M, Leodolter A, Schulte E, Vieth M, Jaspersen D, Labenz J, Meyer-Sabellek W, Stolte M, Malfertheiner P, Willich S. (2002). Quality of life in patients with gastroesophageal reflux disease. Abstracts of the Digestive Disease Week.

[8] Ronkainen JA, Aro P, Storskrubb T, Vieth M, Lind T, Graffner H, Talley NJ, Agréus L. (2002). Prevalence of esophagitis and endoscopy-negative reflux disease in a population. A report from the Kalixandra Study. Abstracts of the Digestive Disease Week.

[9] Mohammed I, Cherkas L, Riley SA, Spector TD, Trudgill NJ (2002). Genetic influences in gastro-oesophageal reflux disease: a twin study. Abstracts of the Digestive Disease Week.

[10] Saeian K, Jean M, Kern M, Knox J, Shaker R (2002). Relationship of body mass index and family clustering with symptoms of gastroesophageal reflux disease among obese individuals. Abstracts of the Digestive Disease Week.

[11] Nandurkar S, Cameron A, Fett S, Zinsmeister A, Locke III GR (2002). Environmental causes of reflux: influence of lifestyle, diet and psychological factors. Abstracts of the Digestive Disease Week.

[12] Cherry J, Margulies SI (1968). Contact ulcer of the larynx. Laryngoscope.

[13] Katz PO (1990). Ambulatory esophageal and hypopharyngeal pH monitoring in patients with hoarseness. Am J Gastroenterol.

[14] Koufman JA (1991). The otolaryngologic manifestations of gastroesophageal reflux disease (GERD): a clinical investigation of 225 patients using ambulatory 24-hour pH monitoring and an experimental investigation of the role of acid and pepsin in the development of laryngeal injury. Laryngoscope.

[15] Deveney CW, Benner K, Cohen J (1993). Gastroesophageal reflux and laryngeal disease. Arch Surg.

[16] Fraser AG (1994). Review article: gastro-oesophageal reflux and laryngeal symptoms. Aliment Pharmacol Ther.

[17] Koufman JA, Cummins MM. The prevalence and spectrum of reflux in laryngology: a prospective study of 132 consecutive patients with laryngeal and voice disorders. August 23, 1994. Available Internet <Jkoufman@bgsm.edu and gpostma@bgsm.edu. Center for Voice Disorders homepage> [Jan. 20, 2001]

[18] Costa HO, Eckley CA, Fernandes AMF, Destailleur D, Villela PH (1997). Refluxo gastroesofágico: comparação entre os achados laríngeos e digestivos. Rev Port ORL.

[19] Shaw GY, Searl JP (1997). Laryngeal Manifestations of Gastroesophageal Reflux before and after Treatment with Omeprazole. S Med J.

[20] Eckley CA, Marinho V, Ruiz WS, Costa HO (1999). O uso da pH-metria esofágica de dois canais no diagnóstico da laringite crônica por refluxo gastroesofágico. Rev Bras ORL.

[21] Ulualp SO, Toohill RJ, Hoffmann R, Shaker R (1999). Pharyngeal Ph Monitoring in Patients with Posterior Laryngitis. Otolaryngol. Head & Neck Surg.

[22] Ulualp SO, Toohill RJ, Gu C, Shaker R (2001). Loss of Secondary Esophageal Peristalsis is not a Contributory Pathogenetic Factor in Posterior Laryngitis. Ann Otol Rhinol Laryngol.

[23] Haggitt RC (2000). Histopathology of reflux-induced esophageal and supraesophageal injuries. Am J Med.

[24] Hanson DG, Jiang JJ, Conley D, Kahrilas P (2000). Role of esophageal pH recording in management of chronic laryngitis: an overview. Ann Otol Rhinol Laryngol.

[25] Eckley CA. Estudo da concentração salivar do fator de crescimento epidérmico em indivíduos com laringite crônica por refluxo laringofaríngeo. São Paulo, 2002. (Tese – Doutorado – Faculdade de Ciências Médicas da Santa Casa de São Paulo)

[26] Ylitalo R, Lindestad P, Ramel S (2001). Symptoms, laryngeal findings, and 24-hour pH monitoring in patients with suspected gastroesophagopharyngeal reflux. Laryngoscope.

[27] Waring JP, Lacayo L, Hunter J, Katz E, Suwak B (1995). Chronic cough and hoarseness in patients with severe gastroesophageal reflux disease. diagnosis and response to therapy. Dig Dis Sci.

[28] El-Serag HB, Lee P, Buchner A, Inadomi JM, Gavin M, Mccarthy DM (2001). Lanzoprazole treatment of patients with chronic idiopathic laringitis: a placebo-controlled trial. Am J Gastroenterol.

[29] Gavazzoni FB, De Ataíde AL, Herrero Junior F, Macedo Filho ED (2002). Esofagite por refluxo e laringite por refluxo: estágios clínicos diferentes da mesma doença?. Rev Bras ORL.

[30] Gill GA, Arthur C, Hampson F, Dettmar P, Moorghen M, Pignatelli M (2002). Characterization of acid and pepsin damaged laryngeal and oesophageal mucosa. Abstracts of the Digestive Disease Week.

[31] Eckley CA, Costa HO (2003). Estudo da concentração salivar do fator de crescimento epidérmico em indivíduos com laringite crônica por refluxo laringofaríngeo. Rev Bras ORL.

[32] Eckley CA, Michelsohn N, Tadokoro CE, Rizzo LV, Costa HO (2004). Salivary EGF concentration in adults with reflux laryngitis. Otolaryngol. Head & Neck Surg.

[33] Nostrant TT (2000). Gastroesophageal Reflux and Laryngitis: A Skeptic's View. Am J Med.

[34] Demeester TR, Johnson LF (1976). The evaluation of objective measurements of gastroesophageal reflux and their contribution to patient management. Surg Clin N Am.

[35] Dent J, Dodds WJ, Friedman RH, Sekiguchi T, Hogan WJ, Arndorfer RC, Petrie DJ (1980). Mechanism of gastroesophageal reflux in recumbent asymptomatic human subjects. J Clin Invest.

[36] Dent J, Holloway RH, Toouli J, Dodds WJ (1988). Mechanisms of lower oesophageal sphincter incompetence in patients with symptomatic gastroesophageal reflux. Gut.

[37] Hirschowitz BI (1991). A critical analysis, with appropriate controls, of gastric acid and pepsin secretion in clinical esophagitis. Gastroenterol.

[38] Bumm R, Feussner H, Hölscher AH, Jörg K, Dittler HJ, Siewert JR (1992). Interaction of gastroesophageal reflux and esophageal motility. Evaluation by ambulatory 24-hour manometry and pH-metry. Dig Dis & Sci.

[39] Fiorucci S, Santucci L, Chiucchiú S, Morelli A. (1992). Gastric acidity and gastroesophageal reflux patterns in patients with esophagistis. Gastroenterol.

[40] Smit CF, Tan J, Mathus-Vliegen LM, Devriesse PP, Brandsen M, Grolman W, Schouwenburg PF (1998). High incidence of gastropharyngeal and gastroesophageal reflux after total laryngectomy. Head Neck.

[41] Delahunty JE, Cherry J (1968). Experimentally produced vocal cord granulomas. Laryngoscope.

[43] Helm JF, Dodds WJ, Hogan WJ, Soergel KH, Egide MS, Wood CM (1982). Acid neutralizing capacity of human saliva. Gastroenterol.

[45] Sarosiek J, Mccallum RW (1995). What Role do Salivary Inorganic Components Play in Health and Disease of the Esophageal Mucosa?. Digestion.

[46] Sarosiek J, Mccallum RW (1995). Do salivary Organic Components Play a Protective Role in Health and Disease of the Esophageal Mucosa?. Digestion.

[47] Sarosiek J, Scheurich CJ, Marcinkiewicz M, Mccallum RW (1996). Enhancement of Salivary Esophagoprotection: Rationale for a Physiological Approach to Gastroesophageal Reflux Disease. Am. J. Gastroenterol.

[48] Marcinkiewicz M, Han K, Zbroach T, Poplawski C, Gramley W, Goldin G, Sarosiek J (2000). The Potential Role of the Esophageal Pre-Epithelial Barrier Components in the Maintenance of Integrity of the Esophageal Mucosa in Patients with Endoscopically Negative Gastroesophageal Reflux Disease. Am J Gastroenterol.

[49] Dawes C (1972). Circadian rythms in human salivary flow rate and composition. J Physiol.

[50] Sonnenberg A, Steinkamp U, Weise A, Berges W, Weinbeck M, Rohner HG, Peter P (1982). Salivary Secretion in Reflux Esophagitis. Gastroenterol.

[51] Helm JF, Dodds WJ, Hogan WJ (1987). Salivary response to esophageal acid in normal subjects and patients with reflux esophagitis. Gastroenterol.

[52] Namiot Z, Rourk RM, Piascik R, Hetzel DP, Sarosiek J, Mccallum RW (1994). Interrelationship between Esophageal Challenge with Mechanical and Chemical Stimuli and Salivary Protective Mechanisms. Am J Gastroenterol.

[53] Costa HO, Mesquita Neto O, Eckley CA (2004). Correlação do pH e volume salivares com sintomas laringofaríngeos. Rev Bras ORL.

[54] Costa HO, Mesquita Neto O, Eckley CA (2005). Is there a correlation between saliva pH and Volume and Reflux Laryngitis?. Dysphagia.

[55] Kongara KR, Soffer EE (1999). Saliva and esophageal protection. Am J Gastroenterol.

[56] Korsten MA, Rosman AS, Fishbein S, Shlein RD, Goldberg HE, Biener A (1991). Chronic xerostomia increases esophageal acid exposure and is associated with esophageal injury. Am J Med.

